# Effects of adjuvant low-dose dexamethasone on recurrence in patients with significant residual chronic subdural hematoma after burr-hole drainage: a single-institution retrospective case-control series

**DOI:** 10.1016/j.bas.2025.104316

**Published:** 2025-07-05

**Authors:** Aleksandar Bojović, Milan Lepić, Stefan Mandić-Rajčević, Sanja Lepić, Svetozar Stanković, Aleksandar Djurdjević, Aleksandra Lokaj, Miloš Božić, Nemanja Rančić

**Affiliations:** a Medical Faculty of the Military Medical Academy, University of Defence, Belgrade, Serbia; b Clinic for Neurosurgery, Military Medical Academy, Belgrade, Serbia; c School of Public Health, Faculty of Medicine, University of Belgrade, Belgrade, Serbia; d Faculty of Medicine, University of Belgrade, Serbia; e Institute of Radiology, Military Medical Academy, Belgrade, Serbia

**Keywords:** Chronic subdural hematoma, Recurrence, Corticosteroids, Low-dose dexamethasone

## Abstract

**Objective:**

Recurrence after surgical treatment remains a significant challenge in the management of chronic subdural hematoma (CSDH). Patients with large postoperative residual collections are at increased risk, and adjuvant dexamethasone treatment appears promising as it may reduce inflammation within residual collections.

**Research question:**

This study evaluates the effectiveness of adjuvant low-dose dexamethasone treatment in reducing recurrence rates in patients with significant residual hematomas (>50 %) after burr-hole drainage.

**Methods:**

A retrospective case-control study was conducted on CSDH patients treated at the Military Medical Academy, Belgrade, from 2016 to 2023. Patients undergoing burr-hole craniostomy with postoperative residual CSDH collection >50 % of the preoperative volume were included. A subset received adjuvant low-dose dexamethasone (initial 2 mg twice daily, tapering over three weeks) decided arbitrarily by the treating neurosurgeon. Primary outcome was recurrence.

**Results:**

A total of 99 patients met the inclusion criteria, with a mean age of 74.1 years. Recurrence was observed in 16 of 70 patients (22.9 %) in the control compared to only 1 of 29 (3.4 %) in the dexamethasone group (OR = 0.12, p = 0.045). The protective effect remained pronounced in the multivariate model, and only a single mild dexamethasone adverse event was observed.

**Discussion and conclusion:**

Adjuvant low-dose dexamethasone significantly reduced recurrence rates in CSDH patients with large postoperative residual collections. These findings suggest that low-dose corticosteroid therapy may be a viable adjunct to surgical treatment, but only in cases at increased risk of recurrence. However, further prospective trials are necessary to confirm these results and establish standardized guidelines.

## Highlights

• Adjuvant low-dose dexamethasone treatment significantly reduced recurrence rate of chronic subdural hematoma (from 22.9 % to 3.4 %) in patients with >50 % residual collection after burr-hole drainage.
• The three-week low-dose regimen (initial 4 mg/day, tapered weekly) was well-tolerated with only one reported mild adverse effect (sleep disturbance).
• Findings support selective postoperative corticosteroid use in high-risk chronic subdural hematoma cases, warranting further prospective validation.

## 1 Introduction

The archaic understanding that chronic subdural hematoma (CSDH) develops as a consequence of a repeated minor head trauma has long ago been replaced by a modern complex pathophysiological mechanism that involves a combination of multiple interconnected processes such as inflammation, neomembranes formation, neovascularization, and fibrinolysis, sometimes following a trivial trauma, which contribute to CSDH formation. The inflammation becomes persistent, leading to capsule formation and progressive fluid accumulation. This response is likely triggered by damage to dural border cells rather than the initial hemorrhage. Inflammatory mediators are consistently found at higher concentrations in CSDH collection and membranes compared to peripheral blood, indicating a localized process which allows it's growth (Edlmann et al., 2017; Pripp and Stanisic, 2014).

Treatment options evolved as well. While surgical evacuation still remains a cornerstone of the CSDH management, and the method of choice for patients with larger hematomas, neurological deficits, or altered consciousness, there is no single surgical technique suitable for all CSDH variants, and the choice of technique is usually based on an individual approach and the surgeon's personal preferences (Laldjising et al., 2020). Medication treatment is reserved for asymptomatic and milder forms of CSDH and requires more frequent clinical and radiological monitoring (Kan et al., 2024/05; Stubbs et al., 2024). Recently, an endovascular approach has been presented as a suitable treatment for a patients with mild-moderate symptoms (Salih et al., 2022).

The most significant complication is recurrence or symptomatic progression despite treatment. The etiology is not fully understood, is multifactorial, and thus difficult to predict. Comorbidities such as hypertension, diabetes, use of anticoagulant/antiplatelet therapy, lower Glasgow Coma Scale scores, and hematoma size are also predictors of recurrence. Postoperative residual collection contains a mixture of irrigation fluid, cerebrospinal fluid, and hematoma fluid (blood and blood degradation products), with a significant amount of proinflammatory cytokines, angiogenic and fibrinolytic mediators, and fibroproliferative markers that facilitate inflammation, angiogenesis, and fibrinolysis. When this residual collection is larger, it creates a good environment for recurrence (Jeon et al., 2023; Holl et al., 2022).

In recent years, numerous studies have investigated different medications, surgical and endovascular options to prevent recurrence (Fakhry et al., 2024). The effects of corticosteroids and their anti-inflammatory action mechanism on CSDH were previously recognized (Holl et al., 2022; Tang et al., 2021; Shrestha et al., 2022; Lodewijkx et al., 2021; Hutchinson et al., 2020), and were even considered powerful enough to avoid surgery. Recent multicentric randomized controlled trials, indicated that high-dose dexamethasone treatment as an initial therapeutic approach, does not eliminate the need for surgical evacuation in most patients, but does have an impact on the recurrence rate (Hutchinson et al., 2020; Miah et al., 2023). Corticosteroids were definitely found to be insufficient as sole treatment modality, and even harmful due to potential complications in the vulnerable CSDH population (Hutchinson et al., 2020; Miah et al., 2023). Nevertheless, others have suggested that corticosteroids might still be beneficial as adjunctive therapy to surgical treatment for CSDH at a lower dose (Tang et al., 2021; Shrestha et al., 2022; Holl et al., 2019), associated with lower recurrence rate (Berghauser Pont et al., 2012), and improved survival (Dran et al., 2007).

Dexamethasone has predominantly been used in high-dose regimens (∼120 mg total, and up to 300 mg) for CSDH within major studies (Hutchinson et al., 2020; Miah et al., 2023; Prud'homme et al., 2016; Agrawal et al., 2024), but failed to replace surgery, showing no functional benefit and increased adverse events. Consequently, underscoring the need for caution with high-dose corticosteroid use and recommendations against their use (Hutchinson et al., 2020; Agrawal et al., 2024; Kolias et al., 2018).

Low-dose dexamethasone regimens (<60 mg total) have not been in the focus of high-quality studies, although individual series have indicated their potential to reduce recurrence rates as a safe adjunct to surgery in patients with CSDH (Shah et al., 2021; Cine, 2023; AbdelFatah, 2023; Mebberson et al., 2020). The aim of this study was to evaluate the effects postoperative adjuvant low-dose dexamethasone treatment might have for a selected group of CSDH patients at an increased risk of recurrence. Namely, to prevent recurrence in patients with a significant (>50 %) residual CSDH after burr-hole drainage.

## 2 Methods

A retrospective case-control study included patients operated on for CSDH at the Military Medical Academy, Belgrade, Serbia, from 2016 to 2023, followed for at least 6 months postoperatively to identify complications or need for reoperation. The study was conducted in accordance with the Declaration of Helsinki, and approved by the Institutional Ethics Committee of the Military Medical Academy, Belgrade, Serbia (protocol number: 13/2024, date: February 28th, 2024). Informed consent was obtained from all subjects involved in the study.

### 2.1 Inclusion criteria

• Computerized tomography (CT)-verified presence of CSDH on one or both sides
• Surgery (single burr-hole craniostomy with irrigation and drainage) performed for CSDH
• Postoperative residual CSDH collection (insufficient evacuation of fluid) with a reduction in CSDH maximum thickness of less than 50 %

### 2.2 Exclusion criteria

• Iatrogenic CSDH (following previous ventriculoperitoneal shunt implantation or craniotomy)
• Corticosteroids contraindications (systemic or severe uncontrolled infections, peptic ulcer, psychiatric disorders)
• Simultaneous presence of other intracranial pathologies (hydrocephalus, shunt, infection, parenchymal hemorrhage or neoplasm)
• Insufficient or missing documentation

### 2.3 Surgical procedure

The surgical procedure was the same in all patients and involved a single burr hole craniostomy with irrigation performed under either local or general anesthesia and drainage for 24–48 h with antibiotic prophylaxis. Computerized tomography (CT) was performed on the first postoperative day to verify the position of the drain, the existence of pneumocephalus and other complications, as well as the residual collection and the patients with a 50 % or more residual collection were included in the study.

### 2.4 Adjuvant dexamethasone treatment

The dexamethasone group of patients received low-dose adjuvant dexamethasone treatment in a fixed three-week regimen postoperatively, while the control group did not.

The decision to administer adjuvant dexamethasone treatment was made arbitrarily by the treating neurosurgeon (some would give adjuvant treatment to all, some to none, and some to those patients at increased risk of recurrence), as there are no guidelines or recommendations on the matter.

The fixed three-week regimen was the same in all patients in the dexamethasone group. It was started 3–5 days after surgery, and involved dexamethasone 2 × 2mg, at 7 a.m. and 1 p.m. for 7 days, followed by a dosage decrease to 2 × 1mg for the second 7 days, and 2 × 0,5 mg for the last 7 days (a total of 39 mg). This dose was chosen due to the assumed low risk of side effects and consistent with natural cortisol release times and locally available dexamethasone tablets of 0.5 mg only.

### 2.5 Data collection

Patient data were obtained from medical records (history of disease, operative protocols, neurological status, laboratory analyses, CT findings) including the baseline patient characteristics (gender, age); preoperative CSDH characteristics (side, bilateral hematoma, hematoma thickness); and neurological findings (GCS, Markwalder), as well as the surgery outcomes (postoperative CSDH thickness, pneumocephalus, and recurrence).

Postoperative pneumocephalus (when present) was classified size-based into: minimal - small, isolated air with no or minimal mass effect; moderate - larger, confluent air collections that might not cause compression of brain tissue but are more substantial in volume; major - large air collections that create a mass effect, displacing or compressing brain structures.

Recurrence is defined as a symptomatic re-accumulation of the CSDH on the side that was surgically treated, with a severity warranting a second surgical intervention (Hutchinson et al., 2020; Miah et al., 2023; Torihashi et al., 2008).

### 2.6 Propensity score matching

The study represents a case-control (retrospective and non-randomized) study to assess treatment effects (use of adjuvant low-dose dexamethasone) on surgical treatment outcomes (recurrence). In such studies, treatment choice is often influenced by subject characteristics, so baseline characteristics of treated subjects often systematically differ from those of untreated subjects. Therefore, systematic differences in baseline characteristics between treated and untreated subjects must be considered when evaluating the effect of treatment on outcomes. For this reason, we used the propensity score method to reduce or eliminate the effects of differences in baseline characteristics between treated and untreated patients (Rosenbaum and Rubin, 1984). The method used was adjustment based on the propensity score, where the outcome variable is regressed to the treatment indicator variable and the estimated propensity score.

In the present data set, the propensity score was calculated using a linear logistic regression model where the outcome was dexamethasone or control group and the predictors were baseline patient characteristics (gender, age) as well as hematoma characteristics (side, bilateral hematoma, hematoma thickness) and neurological findings (GCS, Markwalder). Comparison of score values shows that the study design was set correctly to reduce or eliminate differences in baseline characteristics between the patients.

### 2.7 Statistical analysis

All statistical analyses were performed using IBM SPSS Statistics for Windows, Version 26.0 (IBM Corp., Armonk, NY, USA). Descriptive statistics were computed and presented as mean ± standard deviation (SD) for continuous, and as frequencies and proportions for categorical variables and comparative statistics methods (χ2 test, Student's *t*-test, Fisher's exact test) were used. P-values <0.05 were considered statistically significant.

Univariate logistic regression was performed to assess the association of individual variables with recurrence. Odds ratios (OR) with corresponding 95 % confidence intervals (CI) and p-values were calculated.

Based on the univariate results and clinical relevance, a multivariate logistic regression model was constructed to evaluate independent predictors of recurrence.

## 3 Results

The study included 99 patients (out of 193) with a significant (>50 %) residual CSDH collection who met the inclusion criteria. The mean age was 74.1 years, ranging from 38 to 93 years, and the majority were male (74.7 %). A total of 52 patients (52.5 %) had a preoperative Glasgow Coma Scale (GCS) score of 15, indicating full consciousness. Preoperative Markwalder Grading Scale scores were most commonly grade 2, observed in 49 patients (49.5 %), suggesting moderate neurological impairment. Table 1 summarizes the clinical and radiological characteristics of patients by recurrence status with univariate and multivariate logistic regression.

**Table 1:** Summary of descriptive statistics and univariate and multivariate logistic regression analysis by recurrence status.

	All	Recurrence		Univariate		Multivariate	
		No	Yes				
	N = 99	N = 82	N = 17	OR [95 % CI]	p value	OR [95 % CI]	p value
**Age**	74.1 (9.5)	73.6 (9.8)	76.0 (7.6)	1.03 [0.97; 1.09]	0.351		
**Gender**							
Male	74 (74.7 %)	60 (73.2 %)	14 (82.4 %)	Ref.			
Female	25 (25.3 %)	22 (26.8 %)	3 (17.6 %)	0.58 [0.15; 2.23]	0.432		
**Preop. GCS**	13.91 ± 1.87	13.59 ± 1.91	13.98 ± 1.87	0.91 [0.71; 1.16]	0.44		
**Preop. Markwalder**	2.16 ± 0.94	2.35 ± 1.00	2.12 ± 0.93	1.28 [0.75; 2.18]	0.359		
**Preop. thickness (mm)**	22.24 ± 6.22	23.37 ± 7.04	22.00 ± 6.06	1.04 [0.95; 1.13]	0.408		
**Side**							
Left	63 (63.6 %)	51 (62.2 %)	12 (70.6 %)	Ref.			
Right	36 (36.4 %)	31 (37.8 %)	5 (29.4 %)	0.69 [0.22; 2.13]	0.514		
**Bilateral CSDH**							
No	66 (66.7 %)	54 (65.9 %)	12 (70.6 %)	Ref.			
Yes	33 (33.3 %)	28 (34.1 %)	5 (29.4 %)	0.80 [0.26; 2.51]	0.707		
**Postop. GCS**	14.5 (1.3)	14.7 (0.9)	13.5 (2.3)	0.60 [0.41; 0.86]	0.006	0.65 [0.39; 1.08]	0.093
**Postop. Markwalder**	1.3 (1.3)	1.2 (1.2)	1.8 (1.3)	1.44 [0.97; 2.12]	0.068	1.44 [0.84; 2.48]	0.190
**Postop. thickness (mm)**	15.9 (6.2)	15.0 (5.4)	20.2 (7.9)	1.13 [1.04; 1.23]	0.003	1.15 [1.03; 1.29]	0.013
**Pneumocephalus**							
No	28 (28.3 %)	24 (29.3 %)	4 (23.5 %)	Ref.			
Minimal	37 (37.4 %)	34 (41.5 %)	3 (17.6 %)	0.30 [0.08; 1.13]	0.076	0.45 [0.06; 3.40]	0.442
Moderate	23 (23.2 %)	19 (23.2 %)	4 (23.5 %)	1.02 [0.30; 3.50]	0.975	0.58 [0.09; 3.60]	0.562
Major	11 (11.1 %)	5 (6.1 %)	6 (35.3 %)	8.40 [2.19; 32.23]	0.002	5.47 [0.86; 34.87]	0.072
**Adjuvant low-dose dexamethasone treatment**							
No	70 (70.7 %)	54 (65.9 %)	16 (94.1 %)	Ref.			
Yes	29 (29.3 %)	28 (34.1 %)	1 (5.9 %)	0.12 [0.02; 0.96]	0.045	0.09 [0.01; 1.29]	0.076

**Fig. 1:**
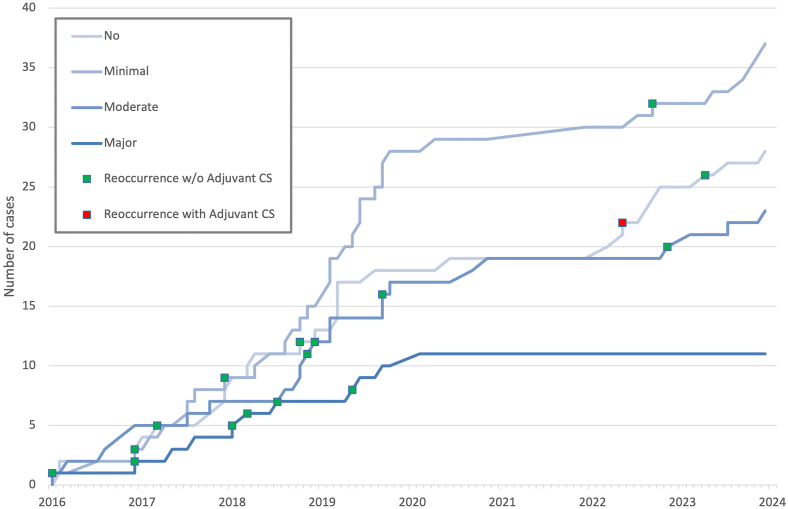
The cumulative inclusion timeline of cases with a significant (>50 %) residual collection by pneumocephalus categories (No, Minimal, Moderate, and Major) and with instances of recurrences (indicated with squares: green - control group; red - dexamethasone group).

There were 17 instances of recurrences (17.2 % recurrence rate) and Fig. 1 depicts the inclusion of patients with different extent of pneumocephalus with emerging recurrences, emphasizing the recurrence rate of only 3.4 % (1/29) in those who received adjuvant treatment compared to 22.9 % (16/70) in control group.

Three factors were significantly associated with recurrence. Lower postoperative GCS scores (mean 13.5 vs. 14.7; OR = 0.60 [95 % CI: 0.41–0.86]; *p* = 0.006), greater postoperative CSDH maximum thickness (mean 20.2 mm vs. 15.0 mm; OR = 1.13 [1.04–1.23]; *p* = 0.003), and the presence of major pneumocephalus (OR = 8.40 [2.19–32.23]; *p* = 0.002) were all significantly associated with increased recurrence risk.

Conversely, adjuvant low-dose dexamethasone treatment therapy was consistently associated with a substantially lower risk of recurrence of chronic subdural hematoma across all analyses. In univariate logistic regression, patients receiving adjuvant CS had 88 % lower odds of recurrence compared to those not receiving it (OR = 0.12, 95 % CI: 0.02–0.96; p = 0.045). Although the association did not reach statistical significance in the multivariate model, the protective effect remained pronounced, with adjuvant CS associated with a 91 % reduction in recurrence odds, but, within a relatively wide 95 % CI: 0.01–1.29, after adjusting for postoperative GCS, Markwalder score, hematoma thickness, and pneumocephalus (OR = 0.09, p = 0.076).

These findings suggest a potential protective role of low-dose adjuvant corticosteroids in reducing recurrence after surgery for CSDH, even if statistical significance was not consistently reached in adjusted models. Moreover, adverse events of dexamethasone use, potentially leading to discontinuation of the adjuvant treatment (such as hyperglycemia, new-onset diabetes, hyperosmolar hyperglycemic state, new-onset psychosis, peptic ulceration or gastrointestinal bleeding, and other upper gastrointestinal symptoms) were not observed. One patient in the dexamethasone group experienced insomnia as an adverse event, but sought no medical attention for it.

## 4 Discussion

The findings of this study suggest that the use of low-dose dexamethasone as a postoperative adjuvant treatment in patients with significant residual CSDH is safe and could reduce the rates of recurrence and sequentially the need for reoperation or endovascular treatment.

Regarding the impact on recurrence rates, the results of present study align with several recent studies indicating that corticosteroids might be beneficial as an adjunctive therapy to surgical treatment for CSDH (Tang et al., 2021; Shrestha et al., 2022; Holl et al., 2019), but in our experience, only in patients at increased risk of reoccurrence. Specifically, our results show that the group at high risk for recurrence that received adjunctive corticosteroid therapy had a lower recurrence rate (3.4 %) compared to those who did not receive therapy (22.9 %).

The pathophysiological basis for the beneficial effects of corticosteroids in CSDH management is likely related to their anti-inflammatory properties. As noted in previous studies, inflammation plays a crucial role in the formation and enlargement of CSDH (Edlmann et al., 2017; Pripp and Stanisic, 2014). Corticosteroids may mitigate this inflammation, reducing the pro-inflammatory factors within the residual CSDH collection that could otherwise trigger further angiogenesis and fibrinolysis, leading to rebleeding or recurrence (Holl et al., 2022). In particular, our study focused only on a portion of patients at high risk for recurrence, particularly those with more than 50 % residual CSDH collection postoperatively. These patients are generally at greater risk for recurrence due to the substantial remaining hematoma, which can perpetuate the inflammatory cycle and prevent CSDH remnants resorption (Jeon et al., 2023).

While our findings suggest a potential benefit of low-dose dexamethasone, the conclusions must be interpreted with caution due to the low number of patients. In the group treated with adjuvant low-dose dexamethasone only one patient (3.4 %) experienced recurrence compared to the 16 (22.9 %) in the control group, but this confirms that the group of patients is indeed at the high risk of recurrence (Jeon et al., 2023).

Postoperative maximum thickness of the CSDH emerged as a consistent and statistically significant predictor of recurrence in both univariate (OR = 1.18, 95 % CI: 1.06–1.31; p = 0.003) and multivariate analysis (OR = 1.29, 95 % CI: 1.03–1.63; p = 0.029). These findings suggest that greater residual hematoma volume is a key factor contributing to recurrence, likely due to insufficient brain re-expansion and ongoing inflammatory activity within the subdural space. Importantly, the effect of adjuvant corticosteroid treatment appeared more pronounced among patients with larger postoperative collections, indicating that anti-inflammatory modulation may be particularly beneficial in this high-risk subgroup. This observation supports the rationale for selective use of corticosteroids based on early postoperative imaging and highlights the need for tailored postoperative management strategies to reduce recurrence risk.

Pneumocephalus could complicate the clinical course and outcomes of CSDH treatment, as it was recognized as an independent predictor of recurrence (Shen et al., 2019), and potentially conflict with the effects of corticosteroid therapy as subdural collection contains air instead of inflammatory factors rich fluid. Pneumocephalus demonstrated a severity-dependent association with recurrence. In univariate analysis, major pneumocephalus was significantly associated with increased recurrence risk (OR = 8.40, *p* = 0.002), with recurrence observed in 35.3 % of patients with major pneumocephalus. Moderate pneumocephalus showed no clear association (OR = 1.02, *p* = 0.975), while minimal pneumocephalus trended toward a lower recurrence risk (OR = 0.30, *p* = 0.076). In multivariate analysis adjusting for other factors, major pneumocephalus retained a strong association with recurrence (OR = 5.47, *p* = 0.072), though statistical significance was attenuated. These findings highlight the disproportionate risk carried by patients with extensive postoperative pneumocephalus and suggest that preventing or minimizing pneumocephalus may reduce recurrence rates after surgery treatment (positioning, air aspiration or oxygen therapy) (Lepic et al., 2021; Chavakula et al., 2020).

The study by Hutchinson et al. reported a general 6.4 % incidence of any infection in patients treated with dexamethasone compared to 1 % in the placebo group, while 1.7 % had surgical site infection (Hutchinson et al., 2020). Adverse events of special interest relevant to the dexamethasone use were not observed in our study. Only one patient developed superficial wound infection and belonged to the group that had not received adjuvant low-dose dexamethasone treatment. On the other hand, one patient experienced insomnia as an adverse effect of dexamethasone but did not seek medical help for it.

Several studies have explored varying dosages and regimens of corticosteroid therapy for CSDH. The standard regimen typically involves a tapering dose over two weeks, starting with 8 mg twice daily (Kolias et al., 2018). However, high-dose therapies of ∼124 mg and up to 300 mg have been found to carry significant risks of adverse drug events (glucose levels and blood pressure increase, sleep deprivation, confusion) (Prud'homme et al., 2016). Our study focused on a low-dose regimen, which, although not extensively studied in high-quality trials, has shown potential benefits and a safe profile (AbdelFatah, 2023).

Despite these promising results, the optimal dosage, timing, and indications for corticosteroid use in CSDH remain to be clearly defined (Yang and Huang, 2017). Further randomized controlled trials are necessary to establish standardized guidelines and confirm the efficacy and safety of low-dose corticosteroids therapy in this context.

The retrospective nature of this study and the non-randomized assignment of treatment pose inherent limitations. The sample size was relatively small in general, especially the dexamethasone group with only 29 patients. The inclusion of only patients at increased risk of recurrence due to a large residual subdural collection contributed to the limited sample, and larger studies are needed to validate these findings. Due to this small number of patients, our methodology employed the propensity score adjustment to account for baseline differences between the treated and untreated groups, following the framework laid by Rosenbaum and Rubin (1984). This approach enhances the reliability of our findings by mitigating selection bias inherent in observational studies, although it could not replace proper randomization. Additionally, the decision to administer corticosteroids was based on the discretion of the neurosurgeon, which could introduce additional selection bias, although we believe it could have improved the outcome recording bias.

## 5 Conclusions

In the present study, in patients with significant (>50 %) residual CSDH after surgery adjuvant low-dose dexamethasone treatment was associated with reduced recurrence rates, but this finding should be taken with caution due to the small sample and study retrospective design. Nevertheless, the results are promising and further prospective studies with larger sample sizes are necessary to elucidate the potential of adjuvant low-dose corticosteroid therapy in patients with a significant residual collection.

## Declaration of competing interest

The authors declare the following financial interests/personal relationships which may be considered as potential competing interests: Milan Lepic reports article publishing charges was provided by Medical Faculty of the Military Medical Academy, University of Defence, Belgrade, Serbia. If there are other authors, they declare that they have no known competing financial interests or personal relationships that could have appeared to influence the work reported in this paper.

## References

[bib1] AbdelFatah M.A.R. (2023). Medical management of chronic subdural hematoma with low-dose dexamethasone: a case series study. NPG Neurologie - Psychiatrie - Gériatrie. 2022/08/29/.

[bib2] Agrawal A., Gupta A., Mishra R. (2024). How dexamethasone affects necessity for surgical intervention for chronic subdural hematoma: systematic review and meta-analysis. Indian J. Neurotrauma.

[bib3] Berghauser Pont L.M., Dammers R., Schouten J.W., Lingsma H.F., Dirven C.M. (2012). Clinical factors associated with outcome in chronic subdural hematoma: a retrospective cohort study of patients on preoperative corticosteroid therapy. Neurosurgery.

[bib4] Chavakula V., Yan S.C., Huang K.T. (2020). Subdural pneumocephalus aspiration reduces recurrence of chronic subdural hematoma. Oper. Neurosurg..

[bib5] Cine H.S. (2023). The use of adjuvant dexamethasone in chronic subdural hematoma after surgery. Cureus.

[bib6] Dran G., Berthier F., Fontaine D., Rasenrarijao D., Paquis P. (2007). [effectiveness of adjuvant corticosteroid therapy for chronic subdural hematoma: a retrospective study of 198 cases]. Neurochirurgie.

[bib7] Edlmann E., Giorgi-Coll S., Whitfield P.C., Carpenter K.L.H., Hutchinson P.J. (2017). Pathophysiology of chronic subdural haematoma: inflammation, angiogenesis and implications for pharmacotherapy. J. Neuroinflammation.

[bib8] Fakhry R., Dirven C.M.F., Moudrous W. (2024). Additional treatment after primary conservative treatment in patients with chronic subdural hematoma-A retrospective study. Brain Behav..

[bib9] Holl D.C., Volovici V., Dirven C.M.F. (2019). Corticosteroid treatment compared with surgery in chronic subdural hematoma: a systematic review and meta-analysis. Acta Neurochir..

[bib10] Holl D.C., Fakhry R., Dirven C.M.F. (2022). Surgery after primary dexamethasone treatment for patients with chronic subdural Hematoma-A retrospective study. World Neurosurg..

[bib11] Hutchinson P.J., Edlmann E., Bulters D. (2020). Trial of dexamethasone for chronic subdural hematoma. N. Engl. J. Med..

[bib12] Jeon G.J., Rim H.T., Lee H.S. (2023). Factors for predicting recurrence after burr hole drainage for chronic subdural hematoma: a retrospective study. Neurosurg. Rev..

[bib13] Kan P., Fiorella D., Dabus G. (2024). ARISE I consensus statement on the management of chronic subdural hematoma. Stroke.

[bib14] Kolias A.G., Edlmann E., Thelin E.P. (2018). Dexamethasone for adult patients with a symptomatic chronic subdural haematoma (Dex-CSDH) trial: study protocol for a randomised controlled trial. Trials.

[bib15] Laldjising E.R.A., Cornelissen F.M.G., Gadjradj P.S. (2020). Practice variation in the conservative and surgical treatment of chronic subdural hematoma. Clin. Neurol. Neurosurg..

[bib16] Lepic M., Mandic-Rajcevic S., Pavlicevic G., Novakovic N., Rasulic L. (2021). Awake surgery in sitting position for chronic subdural hematoma. Acta Neurochir..

[bib17] Lodewijkx R., Holl D.C., Slot K.M. (2021). Effect of steroids as an adjunct to surgical treatment in patients with chronic subdural hematoma. J. Neurotrauma.

[bib18] Mebberson K., Colditz M., Marshman L.A.G., Thomas P.A.W., Mitchell P.S., Robertson K. (2020). Prospective randomized placebo-controlled double-blind clinical study of adjuvant dexamethasone with surgery for chronic subdural haematoma with post-operative subdural drainage: interim analysis. J. Clin. Neurosci. : Off. J. Neurosurg. Soc. Australasia.

[bib19] Miah I.P., Holl D.C., Blaauw J. (2023). Dexamethasone versus surgery for chronic subdural hematoma. N. Engl. J. Med..

[bib20] Pripp A.H., Stanisic M. (2014). The correlation between pro- and anti-inflammatory cytokines in chronic subdural hematoma patients assessed with factor analysis. PLoS One.

[bib21] Prud'homme M., Mathieu F., Marcotte N., Cottin S. (2016). A pilot placebo controlled randomized trial of dexamethasone for chronic subdural hematoma. Can. J. Neurol. Sci..

[bib22] Rosenbaum P.R., Rubin D.B. (1984). Reducing bias in observational studies using subclassification on the propensity score. J. Am. Stat. Assoc..

[bib23] Salih M., Khorasanizadeh M., McMillan N. (2022). Cost comparison for open surgery versus middle meningeal artery embolization in patients with chronic subdural hematomas: a propensity score matched analysis. World Neurosurg..

[bib24] Shah S.A., Mallah F.A., Razaque Mari A., Mirbaher I., Sheikh H.A., Dilbar M. (2021). Role of dexamethasone in recurrent and residual chronic subdural hematoma. Pak. J. Neurol. Surg..

[bib25] Shen J., Yuan L., Ge R. (2019). Clinical and radiological factors predicting recurrence of chronic subdural hematoma: a retrospective cohort study. Injury.

[bib26] Shrestha D.B., Budhathoki P., Sedhai Y.R. (2022). Steroid in chronic subdural hematoma: an updated systematic review and meta-analysis post DEX-CSDH trial. World Neurosurg..

[bib27] Stubbs D.J., Davies B.M., Edlmann E. (2024). Clinical practice guidelines for the care of patients with a chronic subdural haematoma: multidisciplinary recommendations from presentation to recovery. Br. J. Neurosurg..

[bib28] Tang G., Chen J., Li B., Fang S. (2021). The efficacy of adjuvant corticosteroids in surgical management of chronic subdural hematoma: a systematic review and meta-analysis. Front. Neurol..

[bib29] Torihashi K., Sadamasa N., Yoshida K., Narumi O., Chin M., Yamagata S. (2008). Independent predictors for recurrence of chronic subdural hematoma: a review of 343 consecutive surgical cases. Neurosurgery.

[bib30] Yang W., Huang J. (2017). Chronic subdural hematoma: epidemiology and natural history. Neurosurg. Clin..

